# Rabs, Rips, FIPs, and Endocytic Membrane Traffic

**DOI:** 10.1100/tsw.2003.69

**Published:** 2003-09-15

**Authors:** Rytis Prekeris

**Affiliations:** Department of Cellular and Structural Biology, School of Medicine, University of Colorado Health Sciences Center, 4200 E. Ninth Ave., Room 4520, B-111, Denver, CO 80262, USA

**Keywords:** Rab11 GTPase, membrane traffic, transport vesicle, endocytosis, ARF GTPases, cell motility, myosin, molecular motors

## Abstract

Rab GTPases, proteins belonging to the Ras-like small GTP-binding protein superfamily, have emerged as master regulators of cellular membrane transport. Rab11 GTPase, a member of the Rab protein family, plays a role in regulating various cellular functions, including plasma membrane recycling, phagocytosis, and cytokinesis. Rab11 acts by forming mutually exclusive complexes with Rab11-family binding proteins, known as FIPs. Rab11-FIP complexes serve a role of “targeting complexes” by recruiting various membrane traffic factors to cellular membranes. Recent studies have identified several Rab11-FIP complex-binding proteins that regulate distinct membrane traffic pathways.

## INTRODUCTION

Eukaryotic cells compartmentalize biological functions in a series of organelles. In the exocytotic and endocytotic pathways, the unique composition of each compartment is maintained despite the continuous anterograde and retrograde movement of proteins and lipids within the cell. This is accomplished by multiple sorting mechanisms during the formation, transport, and fusion of transport vesicles/tubules with specific target compartments.

Reports from the past several years have revealed new insights into the molecular machinery that regulates the rates and specificity of membrane transport and fusion. Several families of proteins have been identified as key components of transport vesicles/tubules (Fig. 1). These groups of proteins constitute the core of membrane traffic and usually mediate a specific trafficking step. Indeed, coat proteins (e.g., COPs and clathrin) mediate vesicle budding from the donor compartment as well as cargo selection (Fig. 1, step 1). Actin- and microtubule-based molecular motors are responsible for the delivering transport vesicle to its final destination (Fig. 1, step 2). Tethering proteins ensure the docking of transport vesicles to target membranes (Fig. 1, step 3). Finally, SNARE proteins, namely syntaxins and VAMPs, mediate membrane fusion and

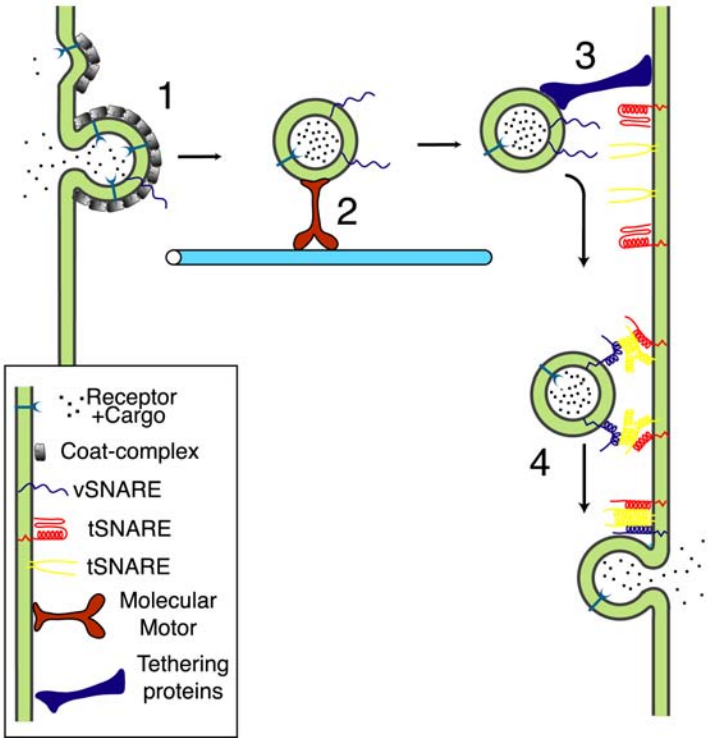

**FIGURE 1.** Transport vesicle formation and fusion model. Coat proteins mediate vesicle budding from the donor compartment as well as cargo selection (step 1). Actin- and microtubule-based molecular motors are responsible for delivering the transport vesicle to its final destination (step 2). Tethering proteins dock transport vesicles to target membranes (step 3). Finally, SNARE proteins, namely syntaxins and VAMPs, mediate membrane fusion and delivery of cargo to the acceptor compartment (step 4). VAMPs are usually present on transport vesicles (vSNAREs), while syntaxins are present on target membranes (tSNAREs).

delivery of cargo to the acceptor compartment (Fig. 1, step 4). For more detailed information about SNAREs, molecular motors, and coat proteins, the reader is directed to other recent reviews[1,2,3,4].

It has become increasingly apparent that while the core machinery is required for membrane traffic, the specificity of membrane and protein transport is determined by a large variety of regulatory molecules that impart the spatial and temporal regulation on vesicle formation and fusion. Several groups of proteins — including tethering factors, Rab GTPases, and even SNAREs — have been suggested to play a role in determining the fidelity of membrane transport. This mini-review will discuss the role of the Rab GTPase family, with emphasis on Rab11, as master regulators of cellular membrane traffic and will illustrate the range of interactions between Rab11 and its binding partners to fulfill its role in regulating the fidelity of membrane traffic.

## RAB GTPASES

The first Rab GTPase, Sec4p, was cloned 2 decades ago as a protein required for secretary vesicle exocytosis in yeast[5]. Since then, the full complement of yeast Rabs (Ypts) and more than 60 mammalian Rabs have been identified[6]. Although the specific functions of most of Rab GTPases remain to be elucidated, it has become clear that Rab GTPase family members are important regulators of membrane traffic. As most small monomeric GTPases, Rabs belong to a superfamily of Ras-like proteins that also include Ras, Ran, ARF, and Rac/Rho GTPases. A common feature of small GTPases is that in the GTP-bound state they can interact with variety of downstream effector molecules. GTP hydrolysis leads to conformational change in the "switch" regions that renders the GTPase unrecognizable to its effectors. A key question in understanding the interactions between Rabs and their effectors addresses the mechanisms by which Rab GTPases specifically bind a diverse spectrum of effectors and how this is regulated by the common structural motif used as a GTP switch. Biochemical and genetic studies have identified several hypervariable regions that determine the specificity of effector binding, including N- and C-termini and the $\alpha3/\beta5$ loop[7]. Indeed, the recently reported structure of Rab3a bound to a putative effector, Rabphilin-3a, revealed that the Rab3a/Rabphilin-3a complex interacts through two main regions[8]. The first consists of conformationally sensitive "switch" regions of Rab3a bound to the $\alpha1$ helix and the C-terminal part of Rabphilin-3a. The second involves the SGAWFF domain of Rabphillin-3a, which fits into a pocket formed by the three hypervariable complementary determining regions (CDRs) of Rab3a (they correspond to the N- and C-termini and $\alpha3/\beta5$ loop). Thus, it appears that the hypervariable Rab CDRs are involved in determining the specificity of effector binding, while the conserved "switch" regions impart GTP dependency and binding. It remains to be investigated whether this paradigm applies to other Rab/effector complexes as well.

Intracellularly, Rab GTPases undergo an intricate cycle of membrane association and cytosolic localization (Fig. 2). The majority of Rab GTPases are post-translationally modified at C-terminus by the addition of two hydrophobic geranylgeranyl groups. This process is mediated by Rab escort protein (REP) and appears to be crucial for Rab targeting to appropriate cellular membranes. After GTP hydrolysis, the Rab is extracted from the membranes by forming a complex with a cytosolic GDP-dissociation inhibitor (GDI). Rab is then recycled from the GDI complex to newly forming vesicles with the help of GDI-dissociation factor (GDF). Finally, guanidine nucleotide exchange factor (GEF) promotes the release of GDP and subsequent loading of GTP, resulting in activated Rab protein. In its GTP-bound state, Rab is then free to interact with its effector molecules. The active state of Rab is finally terminated through the action of GTPase activating protein (GAP), which accelerates nucleotide hydrolysis. The GDP-bound Rab can then go through another round of reactivation.

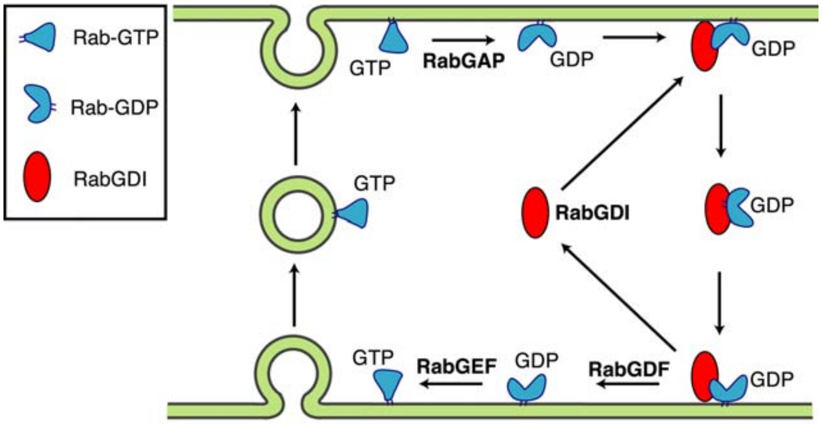

**FIGURE 2.** Rab GTPase intracellular cycle. After GTP hydrolysis, the Rab is extracted from the membranes by forming a complex with a cytosolic GDP-dissociation inhibitor (RabGDI). Rab is then recycled from the RabGDI complex to newly forming vesicles with the help of GDI-dissociation factor (RabGDF). Finally, guanidine nucleotide exchange factor (RabGEF) promotes the release of GDP and subsequent loading of GTP, resulting in activated Rab protein. In its GTP-bound state, Rab is then free to interact with its effector molecules. Rab activity is finally terminated through the action of GTPase activating protein (RabGAP), which accelerates nucleotide hydrolysis. The GDP-bound Rab can then go through another round of activation.

## RAB11 FAMILY GTPASES AND MEMBRANE TRAFFIC

In the past few years, the Rab 11 family GTPases have emerged not only as main regulators of endocytic membrane traffic, but also as modulators of other membrane transport pathways. The Rab11 family is composed of three closely related proteins: Rab11a, Rab11b, and Rab25. Unlike Rab11a and Rab11b that are expressed ubiquitously, Rab25 is expressed almost exclusively in epithelial cell, although its function still remains somewhat unclear[9,10]. The intriguing feature of Rab11 family GTPases is their involvement in regulating a variety of different membrane trafficking steps.

### Constitutive Protein Recycling to Plasma Membrane

Originally, Rab11a was cloned as a small monomeric GTPase that regulates protein recycling from endosomes back to plasma membrane[11,12]. Indeed, Rab11a is enriched in tubular-vesicular structures located in the vicinity of the microtubule organizing center, known as recycling endosomes (RE), where it colocalizes with transferrin receptor (TfR). Overexpression of Rab11 mutants affected the morphology of this compartment as well as inhibited TfR traffic through RE[11,12], suggesting that Rab11 influences both the structural and functional properties of RE.

### Ligand-Induced Receptor and Transporter Insertion into Plasma Membrane

Ligand-induced internalization followed by subsequent return to plasma membrane regulates the function of many receptors and transporters. The effects of many extracellular ligands are often mediated by the family of G protein-coupled seven transmembrane spanning receptors (GPCRs). Although the mechanisms regulating the internalization and recycling of GPCRs have been extensively studied, the endocytic pathways regulating GPCR sorting and exocytosis remain to be fully understood. Data suggest that after entering early endosomes, GPCR are probably sorted away into specialized endocytic organelles distinct from constitutive recycling pathways[13]. Intriguingly, Rab11 was shown to be present on endocytic carriers containing various GPCR, such as CXCR2 chemokine and the M4 muscarinic acetylcholine receptors[14,15]. Furthermore, overexpression of a dominant negative Rab11 mutant (Rab11S25N) inhibits the recycling and sorting of CXCR2 as well as M4 muscarinnic receptors[14,15], suggesting that Rab11 GTPases plays an important part in regulating GPCR sorting and recycling.

Just like for GPCRs, the activity of glucose transporter 4 (GLUT4) is regulated by an extracellular ligand, insulin. Insulin treatment results in movement of GLUT4 from specialized storage vesicles to plasma membrane in adipocytes as well as cardiac myocytes. While the role of Rab11 GTPase in insulin-induced GLUT4 traffic remains unclear, insulin stimulation results in threefold increase in the amount of Rab11 on GLUT4 storage vesicles[16].

### Transport from Trans-Golgi Network to Plasma Membrane

Besides RE, Rab11 was also shown to be present on Trans-Golgi Network (TGN), suggesting that Rab11 family GTPases also play a role in transport on *de novo* synthesized proteins from TGN to plasma membrane. Indeed, overexpression of a dominant negative Rab11a mutant (Rab11aS25N)

led to significant inhibition of the cell surface delivery of vesicular stomatitis virus (VSV-G) protein and caused the accumulation of VSV-G in the TGN[17]. Furthermore, Rab-GDI mediated depletion of membrane-bound Rab11 led to selective inhibition of TGN-to-plasma membrane transport without effecting intra-Golgi transport and endoplasmic reticulum-to-Golgi traffic[17].

## Polarized Protein Traffic in Epithelial Cells

Epithelial cells are structurally and functionally polarized to transport specific molecules selectively and unidirectionally while maintaining a transepithelial barrier. The selective transport is achieved by the partitioning of the plasma membrane into distinct domains: apical and basolateral. These plasma membrane compartments have distinct lipid compositions and contain specific sets of proteins. The fidelity of apical vs. basolateral protein transport is crucial to a variety of epithelial functions. For instance, regulated insertion of $H^+$-$K^+$-ATPase into the apical plasma membrane of gastric parietal cells is responsible for selective secretion of hydrochloric acid into the stomach lumen[18]. Similarly, vasopressin-dependent insertion of aquaporin 2 water channels into apical membranes of renal collecting duct cells increases kidney water retention and consequently results in regulation of blood pressure.

In addition to the targeting of *de novo* synthesized proteins, epithelial cells also are capable of resorting proteins to the correct plasma membrane domain with every round of endocytosis. This requires regulation at multiple membrane traffic steps. Madin-Darby Canine Kidney (MDCK) cells have distinct apical and basolateral compartments and share late endosome and lysosomal compartments[19,20]. In addition, many epithelial cells have a transcytotic pathway, which uses both apical and basolateral endosomes. These pathways have been studied using many different ligands and receptors, but it is still unclear how many regulatory steps and molecules are required to sort and transport compounds to apical or basolateral plasma membrane domains[19,20,21].

Rab11a and Rab25 have both been shown to regulate polarized endosome traffic in epithelial cells. Rab25 is expressed only in polarized epithelial cells and has been implicated in regulating traffic through the subapical population of endosomes[22]. Rab11a is abundantly expressed in polarized cells and localizes to subapical endosomes in both MDCK and Caco-2 cells[22]. Moreover, expression in MDCK cells of Rab11 mutants deficient in GTP-binding inhibits apical recycling and transcytosis of IgA, while it has no effect on basolateral transferrin trafficking[9,22].

Rab11a also was implicated in regulating $H^+$-$K^+$-ATPase–dependent secretion of hydrochloric acid into the stomach lumen. Two lines of evidence suggest that Rab11 plays a major role in regulating the delivery of $H^+$-$K^+$-ATPase to the apical membrane of gastric parietal cells. First, Rab11a is highly enriched in tubulo-vesicular endosomes containing $H^+$-$K^+$-ATPase, with a stoichiometry of one Rab11a per one $H^+$-$K^+$-ATPase[23]. Second, overexpression of a dominant-negative Rab11a mutant (Rab11-N124I) disrupts apical redistribution of $H^+$-$K^+$-ATPase on stimulation[23]. These results highlight the contribution of Rab11 to regulating the traffic of specialized endosomes in a variety of polarized cell types.

## RAB11 FAMILY INTERACTING PROTEINS (FIPS): EFFECTORS OR SCAFFOLDING FACTORS?

Rab GTPases act through recruitment of various "effector" proteins to organelles and vesicles. Thus, much effort has been invested in identifying effector proteins that mediate the role of Rab11. Initially, we identified Rip11, a novel Rab11-interacting protein[24], and subsequently five more related proteins have been identified[25,26,27]. This group of proteins is known as Rab11-family interacting proteins (FIPs). Based on sequence homology, FIPs can be divided into three classes (Fig. 3). Class I FIPs (Rip11a, Rip11b, RCP, and FIP2) contain a C2 domain after N-terminus of the protein. Class II FIPs (FIP3 and FIP4) contain two EF-hands and a proline rich region. Class III has only one member, FIP1, which exhibits no homology to known protein domains. The common feature of all FIP proteins is the presence of a highly conserved, 20-amino acid motif at the C-terminus of the protein, known as Rab11/25 binding domain (RBD) (Fig. 3)[27]. RBD appears to be in predominantly α-helical conformation, allowing for highly

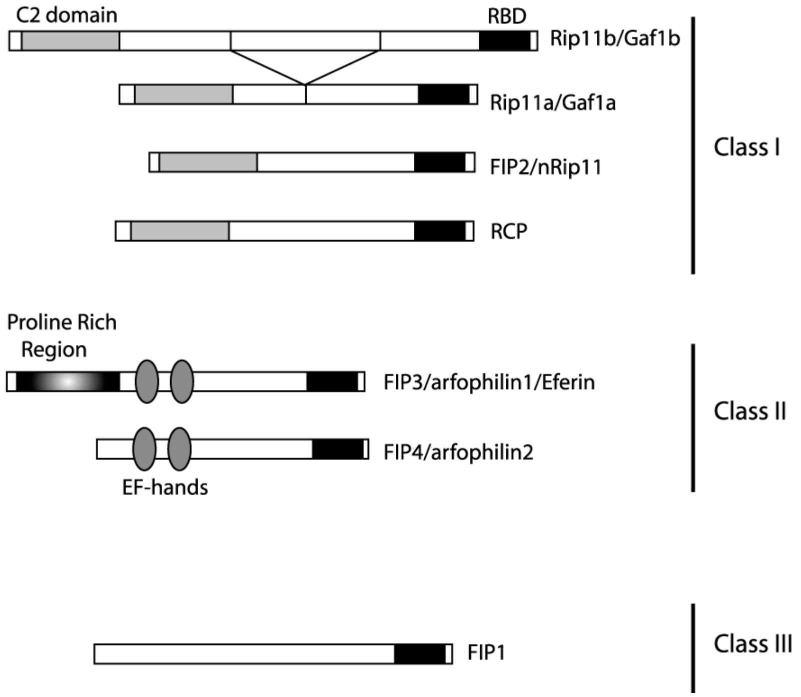

**FIGURE 3.** The structure of Rab11 family interacting proteins (FIPs). Based on their domain structure, all FIPs can be divided into three main classes. Class I FIPs (Rip11a, Rip11b, RCP, and FIP2) contain a C2 domain at the N-terminus end of the protein. Class II FIPs (FIP3 and FIP4) contain two EF-hands and a proline rich region. Class III includes only one member, FIP1, which exhibits no homology to known protein domains. RBD stands for Rab11 binding domain.

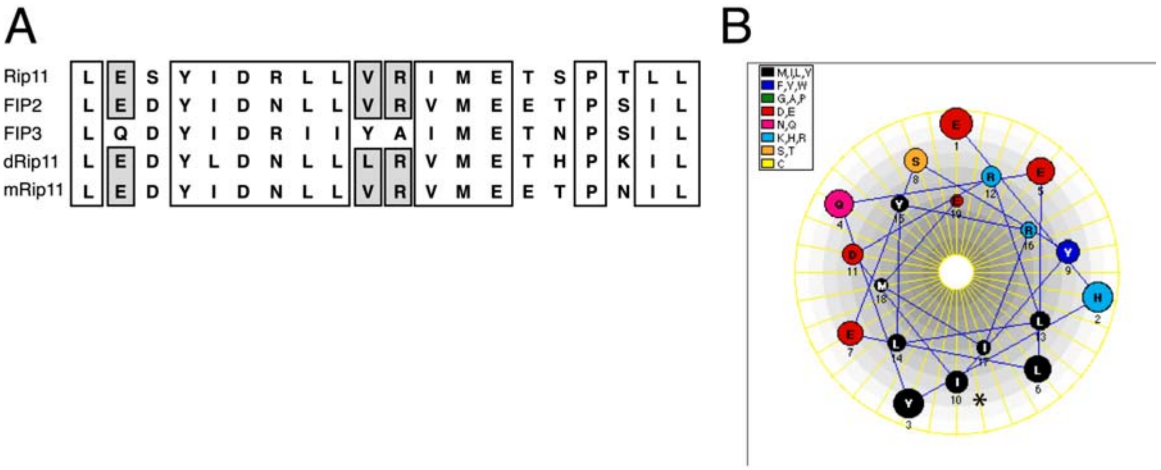

**FIGURE 4.** Sequence and secondary of Rab11 binding domain (RBD). (A) Amino acid alignment of RBD from human Rip11, human FIP2, human FIP3, *Drosophila* Rip11, and murine Rip11-RBD. Boxed regions outline conserved residues. (B) Helical wheel representation of Rip11-RBD (620-639) domain. An asterisk marks the hydrophobic patch required for Rab11 binding.

conserved hydrophobic residues to form a hydrophobic Rab11-binding patch (Fig. 4). Substitution of these hydrophobic residues results in inhibition of Rab11 binding to FIPs[27].

The classical Rab effector proteins are recruited to appropriate organelles through binding their cognate Rab GTPases. Surprisingly, FIP proteins do not apparently require Rab11 for membrane binding[28]. Indeed, overexpression of dominant-negative Rab11 mutant or Rip11 lacking the RBD domain did not disrupt interaction of FIPs with the membranes[28]. Class I FIPs probably bind to endocytic membranes via their C2 domain, which interacts directly with phospholipids[24]. Class II FIPs do not have any *bona fide* membrane binding domains, thus the mechanism mediating FIP3 and FIP4 interactions with endocytic membranes remains to be determined.

What is the function of FIP proteins? One possibility is that they play a role as Rab11 scaffolding proteins. Although Rabs bind to membranes via geranylgeranyl groups attached to C-terminus of the protein, how Rabs are targeted to specific cellular compartments remains unclear. However, it is unlikely that FIPs alone could recruit Rab11 to appropriate endocytic organelles. First, Rip11-I629E mutants that are incapable of binding to Rab11 do not target to appropriate membranes[28]. Second, deletion of geranylgeranylation sites on Rab11 rendered it completely soluble, resulting in a phenotype that could not be rescued by overexpression of FIPs (Prekeris, unpublished data). Thus, it is likely that combinatorial Rab11 and FIP interactions with membranes are responsible for determining the subcellular localization of Rab11-FIP complex. The involvement of Rab11-FIP complex in the regulation of endocytic traffic remains to be fully understood. However, it is tempting to speculate that Rab11 and FIP interaction may serve the role of “targeting complex” that interacts and recruits to organelles a selective group of protein that regulate membrane transport. Recent work from several laboratories has started identifying proteins that interact with specific Rab11-FIP complexes (Table 1).

**TABLE 1**

| FIP Name | Binding Protein | References |
|---|---|---|
| Rip11 | γSNAP | Chen et al., 2001[43] |
| FIP2 | Myosin Vb, Reps 1 | Lapierre, 2001; Cullis et al., 2002 |
| RCP | Rab4 ? | Lindsay et al., 2002 |
| FIP3/Eferin/arfophilin 1 | ARF5, ARF6 | Shin et al., 2001 |
| FIP4/arfophilin 2 | ARF5 | Hickson et al., 2003 |
| FIP1 | ? | — |

## Class I FIP Interacting Proteins

The role of Class I FIPs in regulating membrane traffic is poorly understood. Several proteins interacting specifically with various FIPs have been recently identified (Table 1). Perhaps the best studied is the interaction between Rab11/FIP2 complex and myosin Vb[26,29]. Myosin V proteins are actin-dependent molecular motors that regulate transport of various cellular vesicles[30]. Myosin Va was shown to bind and transport melanosomes through its interactions with Rab27a and melanophilin complex[31,32,33]. Likewise, myosin Vb was recently linked to plasma membrane recycling and Rab11[29]. Yeast two-hybrid screening identified Rab11a and FIP2 as myosin Vb-binding proteins. Furthermore, myosin Vb, FIP2, and Rab11 colocalized to perinuclear recycling endosomes. Consistent with the role of myosin Vb in protein transport through recycling endosomes, overexpression of myosin Vb tail domain inhibited transferrin receptor as well as M4 muscarinic acetylcholine receptor transport to the plasma membrane[14]. While the properties of myosin Vb binding to Rab11a and FIP2 remain to be further characterized, these data suggest that Rab11-FIP2 complex may play a role of the adaptor protein regulating binding of transport vesicles to appropriate motor protein, namely myosin Vb.

In addition to myosin Vb, FIP2 was shown to bind to Reps1, suggesting that FIP2 may regulate clathrin-dependent endocytosis at plasma membrane[34]. Reps1 is an EGF receptor substrate protein containing EH domain. While direct involvement of Reps1 in endocytosis remains to be demonstrated, its close relative, POB1, was shown to regulate AP2-dependent internalization of EGF receptor. More important, FIP2 was shown to bind directly to α-subunit of AP-2, a major clathrin adaptor complex at plasma membrane[34]. The role of FIP2 in EGF receptor endocytosis is also supported by the observation that overexpression of FIP2 inhibits EGF receptor internalization[34].

## Class II FIPs are Dual Rab11 and ARF Interacting Proteins

Emerging evidence suggests that Class II FIPs play a role in coupling Rab- and ADP ribosylation factor (ARF)–dependent membrane traffic. Indeed, Exton and colleagues recently have identified FIP3 as an ARF5 and ARF6 interacting protein[35,36]. Furthermore, FIP4 was also shown to interact specifically with ARF5, but not ARF1, ARF4, or ARF6[37]. Since FIP3 and FIP4 can bind ARFs and Rab11 simultaneously, thus forming a hetero-trimeric complex (Prekeris, unpublished data), it is tempting to speculate that they may serve a role of Rab11 and ARF coupling proteins.

While the role of ARF5 is largely unclear, ARF6 has been actively studied and is known to be involved in several cellular functions such as cell motility, cytokinesis, and phagocytosis[38]. Interestingly, all these functions require the targeted delivery and fusion of large amounts of membranes as well as cytoskeletal rearrangements. The ARF6 role in regulation of cytoskeleton is well understood and is known to involve activation of phospholipase D and Rac/Rho GTPases[38]. In contrast, the role of ARF6 and ARF5 in regulation of membrane traffic remains unclear. At least part of the effect is due to the interactions of ARF5/6 with Golgi localized, gamma adaptin ear homology containing ARF binding proteins (GGA)[2]. GGA along with the adaptin proteins regulate the formation of intracellular transport vesicles and the selection of cargo for incorporation into vesicles. Thus ARF5/6 and FIP3/4 interactions may be crucial in recruiting Rab11 into nascent transport vesicles. Since the full complement of ARF and Rab regulatory proteins are crucial in determining the fate of transport vesicle, FIP3 and FIP4 may be required for transport vesicle targeting. Alternatively, FIP3 and FIP4 may directly regulate transport and targeting of vesicles to their fusion site. In this regard, it has been demonstrated that nuclear fallout protein (Nuf), the putative homologue of FIP3 and FIP4 in *Drosophila melanogaster*, interacts with the microtubule-dependent motor dynein[39,40].

What is the importance of coupling the ARF- and Rab11-dependent pathways? Similar coupling effectors were already reported for other small monomeric GTPases, such as Rab4 /Rab5 as well as ARF/Rho. The coupling effector proteins usually serve a role of proteins integrating the information from various signaling pathways. Perhaps the best example of a pathway requiring both Rab11 and ARF6 is cytokinesis. The formation of a cleavage furrow involves the remodeling of the cytoskeleton as well as delivery of new membranes to the tip of the growing furrow. The cross-talk between the factors regulating both of these processes are likely to play an important role in synchronizing the cytoskeleton remodeling and membrane delivery during cytokinesis. The involvement of ARF6 in regulation of cytokinesis is well documented[41]. Interestingly, Nuf is also required for furrow formation during cellular division in *Drosophila* embryo[39,40]. This hypothesis is also consistent with data demonstrating that Rab11 knock down using RNAi in *C. elegans* results in regression of the cleavage furrow formation as well as localization of FIP4 to the centrosomes in HeLa cells[37,42].

## CONCLUSIONS AND PROSPECTS

This review provided an overview of the most recent developments towards understanding the molecular machinery regulating the function of Rab GTPases in general, and Rab11 GTPase in particular. Identification of a FIP family as Rab11-interacting proteins provided a breakthrough allowing considerable advances in analyzing the role of Rab11. The formation of mutually exclusive Rab11-FIP protein complexes serve as targeting complexes regulating formation, delivery, and fusion of transport vesicles and organelles. Nevertheless, many aspects of Rab11-FIP complex functions remains to be elucidated. They include, but are not limited to: (1) regulation of Rab11-FIP complex formation; (2) differences of FIP binding specificity to Rab11a, Rab11b, and Rab25; (3) identification and characterization of proteins binding to various Rab11-FIP complexes; (4) the nature of cellular processes that are regulated by different Rab11-FIP complexes. The variety of interactions between Rab GTPase and their effector proteins illustrated by analysis of Rab11 reveal the complexity of the cellular machinery used to establish and maintain the fidelity of membrane traffic.

## ACKNOWLEDGMENTS

We thank Drs. Alex Franzusoff and Kathryn Howell for critical reading of the manuscript.
